# The future of automated infection detection: Innovation to transform practice (Part III/III)

**DOI:** 10.1017/ash.2022.333

**Published:** 2023-02-10

**Authors:** Westyn Branch-Elliman, Alexander J. Sundermann, Jenna Wiens, Erica S. Shenoy

**Affiliations:** 1 Section of Infectious Diseases, Department of Medicine, Veterans’ Affairs (VA) Boston Healthcare System, Boston, Massachusetts; 2 VA Boston Center for Healthcare Organization and Implementation Research (CHOIR), Boston, Massachusetts; 3 Harvard Medical School, Boston, Massachusetts; 4 Division of Infectious Diseases, Department of Medicine, University of Pittsburgh, Pittsburgh, Pennsylvania; 5 Division of Computer Science and Engineering, University of Michigan, Ann Arbor, Michigan; 6 Infection Control Unit, Massachusetts General Hospital, Boston, Massachusetts; 7 Division of Infectious Diseases, Department of Medicine, Massachusetts General Hospital, Boston, Massachusetts

## Abstract

Current methods of emergency-room–based syndromic surveillance were insufficient to detect early community spread of severe acute respiratory coronavirus virus 2 (SARS-CoV-2) in the United States, which slowed the infection prevention and control response to the novel pathogen. Emerging technologies and automated infection surveillance have the potential to improve upon current practice standards and to revolutionize the practice of infection detection, prevention and control both inside and outside of healthcare settings. Genomics, natural language processing, and machine learning can be leveraged to improve identification of transmission events and aid and evaluate outbreak response. In the near future, automated infection detection strategies can be used to advance a true “Learning Healthcare System” that will support near–real-time quality improvement efforts and advance the scientific basis for the practice of infection control.

In the past, outbreak identification has partially relied upon human pattern recognition and coincident events. Automated infection detection offers the potential for a systematic approach to identification. However, realizing the full potential of automated detection systems will require the creation of a comprehensive and centralized data collection and analysis repository. Without rich, centralized, and linked data, we will continue to be reliant on human factors and chance to detect rare, but never-miss events, and novel syndromes and pathogens, such as severe acute respiratory coronavirus virus 2 (SARS-CoV-2). Achieving advances facilitated by automated infection detection has the potential to substantially improve patient care and the practice of infection prevention and control but requires significant upfront efforts, multidisciplinary collaborations, and leadership to develop and validate programs.

Early in the human immunodeficiency virus (HIV) epidemic, cases were identified by a pharmacy technician within the US Centers for Disease Control and Prevention (CDC) who received multiple requests for inhaled pentamidine in young men, including one request for re-treatment.^1^ These cases occurred diffusely and across the country, but viewed as a whole they signaled a much larger outbreak and public health crisis. The astute technician noted that these medication requests were far out of proportion with the expected rate for the patient population, and, noting the unusual confluence of requests, reported the cases to her supervisor, thus bringing the outbreak to the attention of the federal government.^1^ At the level of the individual patient, provider, or facility, the signal was difficult to see because isolated cases of even rare conditions often do not raise suspicion of a larger problem. These challenges meant that outbreak identification was slow, and delays limited our ability to deploy infection prevention and public health interventions to mitigate transmission inside and outside healthcare settings. Ultimately, the HIV epidemic led to the introduction of “universal precautions”^2^ in healthcare settings, but implementation of improvements was delayed because the outbreak had not yet been identified.

Decades later, in an early *Morbidity and Mortality Weekly Report*, the Centers for Disease Control and Prevention (CDC) noted that SARS-CoV-2 likely started spreading in the United States in late January or early February 2020 but that current methods of emergency room syndromic surveillance were not sufficiently sensitive to identify the occurrence of community transmission until late February 2020.^3^ In the near future, how can electronic systems be leveraged to improve on current and past processes to improve detection of healthcare-associated infections (HAIs), transmission events, and outbreaks to speed infection prevention responses? How can data generated and analyzed in near real time be used to evaluate infection prevention responses and to advance our understanding about how, where, and when transmission occurs and how to stop it? How many lives would have been saved if we had identified the HIV outbreak 10 years earlier? Or if community spread of SARS-CoV-2 had been identified more quickly and precautions had been deployed more rapidly?

As the electronic health record (EHR) continues to expand, so too does the potential to use automated infection surveillance strategies to improve the practice and evidence basis of infection prevention and control. Systems-level approaches made possible by new and emerging technologies will allow us to rely less on human factors for infection detection and response. Automated infection surveillance strategies may be leveraged to realize a “Learning Healthcare System” in which data collected in near real time are analyzed and applied in clinical settings to improve bedside care and to advance the science of infection prevention (Fig. 1).^4^

**Fig. 1.**
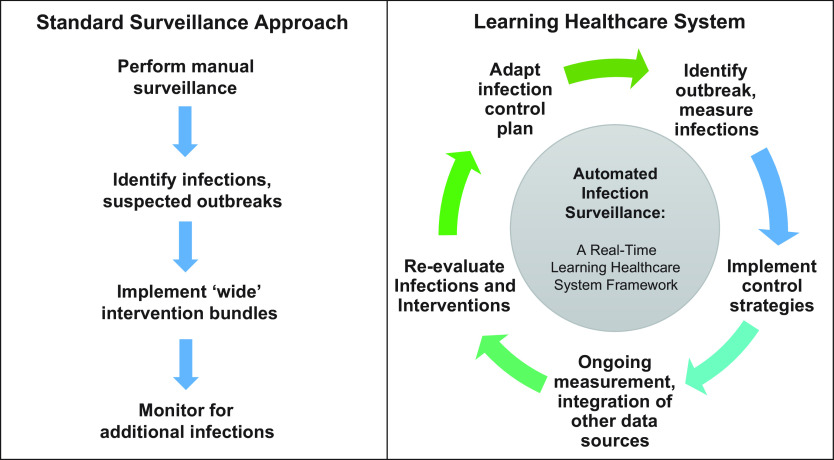
Automated infection surveillance to support real-time, continuous quality improvement and a ‘Learning Healthcare System.’

Innovations likely to be realized in the near future include expansion of infection detection to uncovered clinical areas and HAIs, and application of technological advancements, such as genomics, machine learning, and artificial intelligence, to enhance identification of transmission events and outbreak identification and investigation, both of which are addressed in companion reviews.^5,6^ Leveraging rich electronic data has the potential to expand infection prevention surveillance to support broad quality improvements and to inform the development of predictive, rather than reactive, measurement tools to identify high-risk patients so that they can be specifically targeted for intensive prevention interventions.

In this third review in the series, we focus on how automated infection detection tools may be leveraged to achieve major advances in infection detection, prevention, and control. Practical and economic considerations for supporting these innovations are also briefly discussed.

## Automated infection detection to support a true learning healthcare system

Although challenges remain, the prospect of automated infection surveillance strategies also brings promise (Table 1).^5^ As the EHR continues to expand and real-time informatics capabilities improve, automated infection detection can be integrated into a larger continuous quality improvement system. Data generated in real time can be used to inform decision making and infection control policies, which could then be serially adapted as part of a true learning healthcare system (Fig. 1).^4^ Examples of how real-time automated surveillance could be applied to improve the long-term practice of infection prevention and improve the evidence basis for infection prevention interventions follow.

**Table 1.** Future Opportunities and Potential for Automated Infection Surveillance to Improve the Practice of Infection Prevention

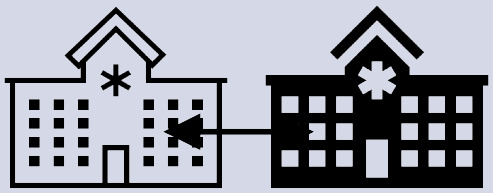	Link automated surveillance across different institutions and healthcare systems to improve data capture and speed outbreak identification and response
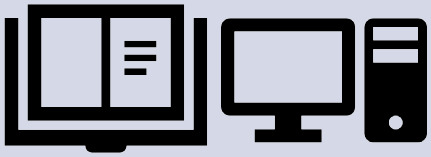	Natural language processing to extract data from clinical documentation to support surveillance activities
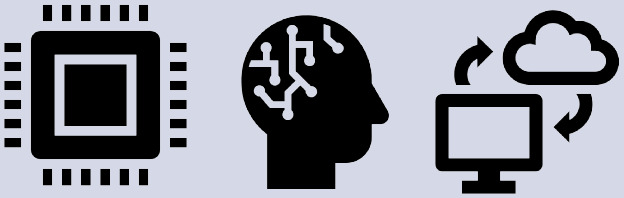	Artificial intelligence to review imaging, pathology results, and to identify patterns and syndromes
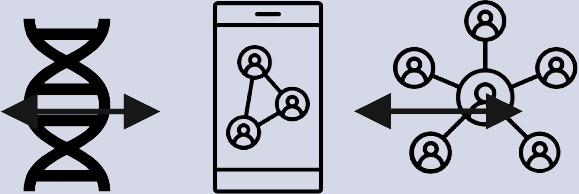	Linkage of genomics and epidemiologic data to identify possible transmission events, including single transmission events, clusters, and outbreaks
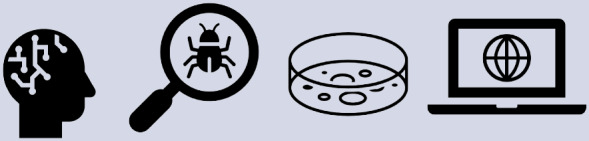	Artificial intelligence for outbreak detection and clinical risk prediction
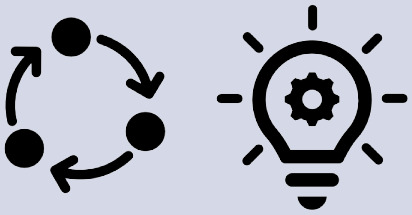	Continuous quality improvement and ongoing assessment of infection prevention and control plans

### Near–real-time evaluation of infection prevention response effectiveness

A major challenge in the practice of infection control is that when an increase in potentially preventable infections is identified, a bundle of interventions is developed to address the problem. Over time, the focus of the intervention improves, whether it be incidence of central-line–associated bloodstream infections or system-wide *Clostridioides difficile* rates. Because of the rarity of events and the natural tendency for regression to the mean, it can be difficult to determine whether the interventions deployed were effective, specifically which bundle elements were effective and should be retained and which were ineffective and therefore an inefficient use of resources. The learning healthcare system framework provides a path for continuous re-evaluation of infection prevention interventions and for generation of real-time data to inform the impacts of implementing (or deimplementing) different interventions. Thus, real-time electronic data tools represent major opportunities for advancing the science of clinical infection prevention practice, for improving care, and for promoting evidence-based deimplementation of ineffective interventions.

## Combining genomics, electronic data, and machine learning to improve outbreak detection and response

### Whole-genome sequencing

Traditional surveillance methods of detecting suspected outbreaks relies on pathogen similarity. Often, isolate antibiograms are compared when an outbreak or transmission is suspected. However, this approach has limited sensitivity and specificity. Sensitivity can be limited due to genetically related pathogens having different antibiograms, and specificity can be limited due to distinct pathogens with similar antibiograms. This discordance can create noise and misclassification when using traditional surveillance tools and traditional epidemiologic associations to detect and evaluate outbreaks.

Genomic methods offer the ability to discriminate whether a common source exists between isolates and therefore whether transmission of a pathogen has likely occurred.^7^ Once reserved for only investigations of major outbreaks, genomic tools are becoming increasingly more common in infection prevention practice. Whole-genome sequencing (WGS) has replaced pulsed-field gel electrophoresis as the gold standard in genomic typing. Using WGS, investigators can show genetic relatedness of pathogens by comparing individual mutations in their genomes.

When is the best time to deploy WGS? Given historically high costs and limited availability, the traditional use case in infection prevention has been in response to suspected transmission events or to confirm or refute the presence of an outbreak after it has already occurred. Often, an outbreak investigation is initiated by infection prevention after a cluster of cases is identified or after questions are raised by a clinician. An infection preventionist (IP) may create line lists of suspected patients involved, perform audits, generate hypothesis, and initiate an intervention. Only then would WGS be applied to identify which isolates are part of an outbreak. However, such an approach is error prone, often misidentifying suspected transmissions and outbreaks^8^ and failing to identify true outbreaks.^7^

Decreasing costs of WGS and wider availability of the technology through public health, academic, and commercial laboratories has enabled some institutions to create WGS surveillance programs and/or to perform sequencing of isolates as they are cultured to detect or rule-out transmission events. These systematic approaches to applying the technology overcome the limitations of reactive sequencing and move away from relying on clinicians and epidemiologic associations to detect outbreaks. Although very promising, systematic applications of WGS have not yet been widely adopted in real-time settings and most of the data about their effectiveness comes from retrospective research investigations.^9^

### Genomics and automated infection detection

WGS can augment automated infection surveillance tools that aim to detect outbreaks and transmission events. Tools such as WHONET-SaTScan use geotemporal aspects and antibiograms to automatically detect potential transmission events. Using the EHR, such tools identify pathogens with similar antibiotic sensitivities that were collected from similar locations and within a relevant timeframe. They can be used to estimate a probability of a transmission event or outbreak. Although it is intriguing to improve upon less systematic approaches to identify transmission events and clusters, the application of the geotemporal approach in a healthcare setting has a high false-positive rate that must be considered before it is implemented.^10^ For example, one study applying the WHONET-SaTScan detected 168 “outbreaks,” of which only 6 (3.5%) were considered to be true transmission events. The geotemporal clustering approach can also fail to identify transmission events, particularly those that occur during procedures (eg, from shared contaminated devices) that are performed in separate locations or on separate days and are therefore not detected. Augmenting geotemporal clustering with WGS surveillance could improve upon epidemiologic linkage alone to improve the positive predictive value of outbreak detection. Once identified, EHR algorithms could then be applied to determine potential routes of transmission to facilitate outbreak investigation and response.

In addition to its utility as a standalone tool for improving evaluations of potential transmission events, a WGS surveillance program coupled with automated infection surveillance tools could significantly assist infection prevention efforts in outbreak investigations. Under current typical circumstances, an infection prevention investigation requires intensive chart review of potentially involved patients. Under a hybrid approach that leverages clinical and genomics data, automated algorithms could extract rich EHR data and instantly analyze it to identify epidemiologic linkages and potential routes of transmission. Previous studies deployed this hybrid approach using a case–control design applied by a machine learning (ML) algorithm to identify associations that warrant additional evaluation. In these studies, cases were defined as all genetically related patient isolates detected by WGS surveillance and controls were all other patients in the hospital.^11,12^ The ML algorithm was able to successfully identify plausible routes of transmission for many of the outbreaks, which limited the need for an intensive, manual chart review process and allowed for a more streamlined outbreak investigation. An additional benefit was the ability of the ML algorithm to detect potential routes of transmission that might otherwise be missed by a fully manual case review process.

### Technological barriers and realities of machine-learning algorithms

Although retrospective studies are intriguing, these advanced data science strategies have not been widely adopted for real-time, operational use. Theoretically, a major benefit of such informatics strategies is the lack of reliance on human resources. However, the reality is that such ML algorithms require labeled training data before they can be applied. Creation of training data sets requires substantial input, either based on data from earlier outbreaks that have been manually adjudicated or from infection prevention experts providing manually reviewed cases from a current investigation. In addition, ML algorithms must also undergo a validation step with a data set that is separate from the training set. The validation data set allows investigators to test the reliability and accuracy of the algorithm in a setting not biased by its training data.

For example, a ML algorithm to detect transmission events in possible outbreak investigations was trained using retrospective data from well-defined outbreaks with known routes of transmission.^13,14^ In the example of the outbreak investigation algorithm, the investigators applied their tool on subsequent WGS surveillance data and validated its findings on how the outbreaks were transmitted.^11^ In the future, once algorithms are trained and validated with historical data, some of these current challenges may be mitigated; however, ongoing maintenance and revalidation will be required to support these innovations.

### Advancing the science of infection prevention by improving identification of clinical trials eligibility and facilitating outcomes assessments

Studies in other fields suggest that automated infection surveillance systems can be used to improve identification of patients eligible for clinical trials and to improve enrollment.^15,16^ Although this approach has not been used in infection control, early identification of patients with HAIs through automated infection surveillance processes provides an opportunity to develop and rapidly test different interventions and to assess their efficacy. After enrollment in a clinical trial, automated surveillance tools can be used as an objective measure of outcome assessment, potentially increasing clinical trial access by reducing the need for specific research expertise at participating centers. Fully automated surveillance of surgical-site infections to compare the effectiveness of oral antibiotics versus standard of care on outcomes following colorectal surgery using National Surgical Quality Improvement data is already underway.^17^ Thus, advancing electronic detection strategies may not only improve case detection and reproducibility but may also be leveraged to advance the state of the science underpinning infection control practice.

### Early warning systems and detection of rare and late healthcare-associated infection transmission events

Current HAI detection systems are inherently focused on near-term outcomes, and although some HAIs may cause limited harm early, substantial and potentially preventable harm may occur many years in the future. Although not currently possible, an artificial intelligence system with broad access to longitudinal EHRs across systems (and ideally genomics data) might be able to identify infections with a longer asymptomatic period early in the disease course or the transmission of novel infectious syndromes. For example, SARS-CoV-2 was not identified until substantial community spread had already been occurring for some time.^3^ Early identification and pre-emptive treatment could avert adverse impacts and could facilitate rapid deployment of infection prevention resources to areas of greatest need.

Within genomics, an example of a centralized data system is PulseNet, which was created by the CDC and combines greater than 80 governmental laboratories across the United States.^18^ Each individual laboratory sequences high-impact, potentially foodborne-related pathogens and submits the data to the CDC. This approach enables the CDC to detect multistate outbreaks that would have otherwise been undetected by individual hospitals or laboratories, which facilitates the public health response.

As EHRs are increasingly linked across healthcare systems, standardized and automated surveillance tools can also be used as part of an early warning system to detect seemingly unrelated clusters earlier, to allow for planning and implementation of interventions to avert additional infections, and to improve upon case reporting that is detected by frontline clinicians alone. For example, prior to implementing an automated infection surveillance strategy for adenovirus-induced hepatitis in children, the New York City Department of Health and Mental Hygiene rarely received reports of the illness. In April 2022, they initiated an automated infection surveillance system of emergency department visits that searched for certain *International Classification of Disease, Tenth Revision* (ICD-10) administrative codes and key words. Through this simple alert system, the health department were informed of ∼5 potential cases per week. These reports were then reviewed by disease investigators to determine whether the patient met the criteria for a patient under investigation (PUI). Within a few months, at least 10 PUIs had been identified in New York City (personal communication).

Advanced detection technologies may be especially useful for identifying extremely rare but “never miss” events such as HAIs that typically manifest many years after exposure (eg, HCV transmissions during endoscopies)^19^ or HAIs that occur outside the healthcare system where the exposure occurred (eg, CRE transmissions from outpatient endoscopies that manifest as sepsis). Each of these scenarios has its own identification challenges; however, all are characterized by difficulties linking an event to the relevant exposure for a variety of reasons, whether that be time or follow-up care in another setting.

For example, viral infections, such as hepatitis C virus (HCV) are rarely transmitted through endoscopy procedures. During a large cluster of HCV infections in New York City,^20^ an outbreak was identified and reported due to a confluence of events: a high rate of acute HCV infections leading to inpatient hospitalizations at 3 different hospitals on the same weekend, simultaneous with the endoscopist who performed the procedures being on call at all of them. If the endoscopist performing the procedure had not happened to be covering all 3 hospitals, and if there had not happened to be relatively high rate of acute HCV infections leading to hospitalization, the cluster would have gone undetected, and practices that led to the infections would have continued. A more reliable approach—not dependent upon human factors— would involve automated surveillance to identify any acute hepatitis case within a specified period after endoscopy to prompt additional case review and evaluation. If this strategy is to be effective, automated infection surveillance across inpatient and outpatient facilities is necessary to allow linkage between the exposure and subsequent events. Ideally, genomics data would also be available to improve accuracy of case linkage, as was completed during the evaluation of several clusters of HBV and HCV infections following myocardial perfusion scans in a cardiology clinic.^21^

### Leveraging EHR data to predict rather than measure

Current IPC surveillance activities focus on infection detection. Retrospective measurement is helpful for identifying potential areas for quality improvement and for measuring the impact of new prevention programs. However, retrospective measurement is limited in its ability to prevent infections. Infection prevention based on *prediction* rather than *detection* has great potential in reducing HAI incidence. In recent years, technological advances in the EHR and data science have brought real-time predictive modeling into current clinical practice. Such tools have been used to predict clinical deterioration, sepsis onset, and more.^22–25^ Predictive models could be used to identify patients at particularly high risk of developing HAIs and to target prevention strategies.^26^ Such tools could also be used to predict complicated or severe disease, enabling early intervention, and improving patient outcomes.^27^

## Cost and resource considerations

Prior investigations suggest that automated infection surveillance strategies have the potential to save substantial infection control practitioner personnel time.^28,29^ This time can be allocated to other quality-improvement related efforts, which may yield benefits and cost savings in other ways. Although theoretically appealing as a strategy for improving efficiency and reducing workload, developing and validating automated infection surveillance tools requires substantial upfront and ongoing investments and possible diversion of resources away from other IPC activities. When considering the costs of automating infection surveillance, all costs—not just the costs to the infection prevention and control department—must be considered. Programming and operational costs may be significant, particularly if frequent updates are needed due to definition changes, additions to the scope of surveillance, or changes in practice patterns or templates that impact the predictive value of electronic measurement tools. In addition, portability and reproducibility across systems cannot be assumed. Although much of the prior work has focused on infection preventionist time, application of implementation science principles for evaluating programmatic costs, such as the Cost of Implementing New Strategies (COINS) framework,^30^ would help capture the true costs of automated infection surveillance program. Understanding the true costs of automation is important for developing infection prevention budgets, for communicating with hospital leadership, and for allocating resources both in the development and maintenance phases of the project.

### Conclusions

In conclusion, automated infection detection offers significant promise for expanding HAI prevention beyond traditional boundaries. Leveraging informatics and genomics has the potential to identify novel transmission patterns and to identify outbreaks earlier and with more precision than current methods. Emerging technologies are also creating opportunities to achieve a true learning healthcare system in which real-time data are used to inform and improve bedside clinical care.

The HIV epidemic in the United States was identified in part due to a cluster of unusual medication orders that were all processed by the same individual at the CDC. In all cases, the patients had what was later found to be late-stage disease, indicating substantial spread for years, or even decades, before the disease was identified. A cluster of HCV infections was identified because one doctor happened to be on call at 3 different hospitals concurrent with admissions for a rare manifestation of acute infection. The key to outbreak detection in both instances was data collection and review by a single, central source. However, such centralized review processes are not standard, and in both examples, the outbreak was identified due largely to chance.

Automated surveillance offers an opportunity to improve on such error-prone processes, but only if the relevant data are collected, integrated, and analyzed in a central repository. If electronic records were linked, could artificial intelligence have identified the HIV epidemic with a smaller number of cases, potentially allowing more rapid response and earlier implementation of universal precautions? Could HCV clusters be identified through random genomic sequencing to link cases and automated intelligence used to conduct a “rapid” case-control study and identify potential sources?

Realizing the full potential of data science to improve HAI detection and improve clinical care will require better integration of EHRs across health systems, close collaboration across disciplines, and substantial investment of resources. Future advancements have the potential to revolutionize HAI prevention efforts and to further the science of infection prevention and control.
